# Prevalence of Aggressive Behavior Toward Fellows, Residents, and Nurses at a Tertiary Care Hospital in Riyadh, Saudi Arabia

**DOI:** 10.7759/cureus.24142

**Published:** 2022-04-14

**Authors:** Faaezuddin Syed, Mohammad Sajid Mithani, Fadwa Abu Mostafa, Areej Alfattani, Joumana Al Messharawi, Hanan Al Ghammas, Dhafer Al Amri, Abdulaziz A Binzaid, Sami Almustanyir

**Affiliations:** 1 College of Medicine, Alfaisal University College of Medicine, Riyadh, SAU; 2 Quality Department, King Faisal Specialist Hospital and Research Centre, Riyadh, SAU; 3 Biostatistics and Epidemiology Department, King Faisal Specialist Hospital and Research Centre, Riyadh, SAU; 4 Adult Cardiology Department, King Faisal Specialist Hospital and Research Centre, Riyadh, SAU; 5 Medicine Department, King Faisal Specialist Hospital and Research Centre, Riyadh, SAU

**Keywords:** fellows, residents, nurses, aggressive behavior, health, burnout, job satisfaction, mental health, wpb, workplace bullying

## Abstract

Background

Workplace bullying (WPB) is a form of mistreatment toward an individual manifested by physical, verbal, or indirect aggression. Affected victims display a wide range of signs and symptoms that impact their health. This study aimed to investigate the prevalence of aggressive behavior toward healthcare workers and its effects on job satisfaction, general health, and mental health.

Methodology

An online survey comprising a revised version of the Negative Acts Questionnaire-Revised (NAQ-R) was distributed to the fellows, residents, and nurses working in a tertiary care hospital. The survey collected information regarding the group’s demographics and their exposure to WPB encountered in the work environment while maintaining confidentiality. Survey results were analyzed using SPSS Statistics version 25 (IBM Corp., Armonk, NY, USA).

Results

Among the 339 participants who filled the survey, 53% of healthcare practitioners in different services had experienced some form of WPB. Among the targeted group, it was noted that female gender (50%), age between 31 and 41 years (57.03%), nurses (51.98%), non-Saudi practitioners (41.94%), and those working in inpatient settings (49.74%) were the most commonly affected individuals in the medical facility. Furthermore, higher bullying prevalence was correlated with lower job satisfaction and mental health levels.

Conclusions

Age, gender, job, and nationality were factors associated with increased susceptibility to WPB. WPB in any facility is an unfortunate event, especially in a healthcare setting. It affects health practitioners by decreasing job satisfaction, jeopardizing health, and increasing the risk of harm to patients. WPB will eventually have a negative impact on the medical facility and the healthcare sector. Hence, hospital administrations should be alarmed about the rise in WPB, and adequate measures must be taken to deal with the root cause of the problem.

## Introduction

The overall performance of a healthcare facility depends on the quality of the service provided by an individual healthcare practitioner working in the facility through an environment of interactive and supportive behavior [1]. Therefore, it is crucial that the practitioners working in a healthcare facility be in their best physical and mental health. Threats to the staff’s physical and mental abilities will lead to a decline in individual performance and will ultimately reflect upon the care provided by the facility. Workplace bullying (WPB) is one such factor recognized to affect medical conduct and decrease personal efficiency.

According to the World Health Organization (WHO), WPB is defined as mistreatment, characterized by repeated, persistent, and long-term exposure of an individual to physical and emotional aggression or abuse. The cause of WPB is multifactorial and can be physical, verbal, or even indirect. Verbal bullying includes inappropriate or abusive and offensive language, taunting, embarrassing, defaming, and sexist comments, among others. Whereas physical assault can be in the form of pushing, hitting, spitting, and mocking, to name a few [1-3], indirect bullying can involve removing a practitioner from a position, project, or even the workplace by the perpetrator [4].

The prevalence of WPB reported by many cross-sectional studies highlights the rise of physical and verbal abuse in the healthcare sector among different positions. Clinicians, fellows, residents, and nurses have been a target of widespread WPB. Verbal abuse has been reported as the primary cause of psychological violence, followed by physical and sexual abuse [2,5,6]. A study published by WHO reported that 39.5% of the respondents in Brazil had experienced verbal abuse. Further, 32.2% of the respondents in Bulgaria, 52% in South Africa, 47.7% in Thailand, and up to 67% in Australia were victims of similar bullying [7]. Previous studies have reported that the prevalence of WPB depends on the following characteristics of the victim: gender, age, race, nationality, presence of disabilities, and educational level [2,5,8]. The main perpetrators of WPB have been reported to be patients, their families/relatives, healthcare practitioners, managers, superiors or seniors, administrators, and supervisors [2,8-12].

WPB poses a significant impact on the normal operations of medical professionals. The ability to endure these offenses depends on the culture and virtuousness displayed on individual and community-based grounds. Chronic tensed and stressed situations in the workplace have been associated with an increase in sick leaves and absenteeism [1]. Moreover, individuals who have been a target of different forms of WPB have reported a broad spectrum of signs and symptoms. These include psychological and somatic complaints such as anxiety, depression, low self-esteem, decreased self-control, burnout, musculoskeletal ailments, higher cardiovascular disease risk, recreational drug abuse, and suicidal attempts. In the long term, WPB is associated with increased susceptibility to performance decline, vulnerable job positions, and increased professional staff turnover [2,5,10,13,14].

The abnormal functioning of medical staff has a higher probability of projecting self-emotions onto the care of patients. Lack of communication, improper protocol adherence, and positive withholding behavior substantially increase the risk of medical errors and jeopardize patients’ safety and care [2,10,15,16].

Although extensive research has been done on this topic to address the prevalence, impact on healthcare officials, and determine the effect on patients, resolving the issue of WPB in the healthcare environment has always been a significant concern. According to the literature, the incidence of WPB is often underreported by the victims in the healthcare facility [2]. This further accentuates the need for prompt recognition of the problem, identifying potential threats, and developing strategies and schemes to aid in early resolution. This study aims to determine the factors associated with WPB, the high-risk groups, and the effect of WPB on medical professionals’ health at a tertiary care hospital in Riyadh, Saudi Arabia, through a cross-sectional method.

## Materials and methods

Study design, setting, and participants

A cross-sectional study was conducted among healthcare workers at a tertiary care hospital located in Riyadh, Saudi Arabia. The total bed capacity of the hospital is 1,934 and manages over 10,000 outpatient visits annually, of which the majority of patients are referred from other hospitals in the country. The hospital encompasses more than 14,650 employees from more than 67 different nations. In general, the healthcare sector in Saudi Arabia is heavily influenced by expatriates, decreasing the proportion of Saudi healthcare professionals to 29.5% physicians and 36.7% nurses, with the Saudis to expatriates ratio of nearly 1 in 3. These numbers are thought to arise due to a severe shortage of the Saudi healthcare workforce [17].

In this study, a completely anonymous survey was distributed and submitted online voluntarily. The survey was sent to a targeted group of fellows, residents, and nurses with varying experience levels and departments, from different backgrounds and nationalities, working full time at the healthcare center. The participants were assured that the survey results would be kept confidential and presented in a way that would maintain their confidentiality. The survey aimed to collect information from fellows, nurses, and residents during different years, working at the hospital, concerning their exposure to aggressive behaviors and WPB in their work environment.

Data collection tool

The survey was distributed online to the target population group with proper instructions. Filling out the survey and submitting it was considered their consent to participate in the study and to include their responses in the analysis. The survey was divided into the following three parts: (1) Demographics: this included the job/duty of the participants (nurse, resident, or fellow), sector they work in (inpatient or outpatient), medical department (different departments), clinic (different clinics), residency year (R1, R2, R3, and R4 for residents), length of service (in years), age (in years), sex (male or female), nationality (different countries), and marital status (single, married, divorced, or widowed). (2) 23 sets of questions to assess participants’ potential WPB exposure (on a five-point scale: never, occasionally, monthly, weekly, daily). (3) Three sets of questions to determine participants’ job satisfaction (on a five-point scale: very satisfied, reasonably satisfied, a little satisfied, dissatisfied, very dissatisfied) and physical and mental health (on a five-point scale: excellent, good, fair, poor, very poor). Filling out the survey was completely voluntary with no impact on the participants’ quality and functioning of their job had they denied participation. The responses were anonymous and kept confidential for the privacy of the participants. Data are analyzed and presented in the results section without including individual responses.

Data analysis

Exposure to WPB was assessed using a revised version of the Negative Acts Questionnaire-Revised (NAQ-R), which is the most widely used tool for assessing WPB [18,19]. It consists of 22 items with responses marked on a five-point scale ranging between specific temporal indicators: never, now and then, monthly, weekly, and daily. NAQ-R is a valid and psychometrically sound measure to examine WPB, as assessed by a large study in India (N = 1,053). The overall NAQ-R scale ranges from 22 to 110. The respondents’ data are classified into three categories based on the two cutoff points used, reflecting their exposure to WPB. Below 33 is classified as never bullied, 33-45 is classified as occasionally bullied, and above 45 is classified as severely bullied [20].

Absolute values and percentages were calculated for qualitative variables. The chi-square and Fisher’s exact tests were used to compare the means of participants’ sociodemographic and work-related characteristics. SPSS Statistics version 25 (IBM Corp., Armonk, NY, USA) was used for data processing. Proper adherence to Strengthening the Reporting of Observational Studies in Epidemiology (STROBE) guidelines was observed throughout the research process [21].

Ethics approval

The study was submitted by the Quality Management Division Research Team and approved by the Ethics Review Committee (ERC) with reference number RAC#2181 119.

## Results

Participant characteristics

A total of 339 participants responded to the survey and participated in the study. The sample consisted of 244 (72%) females and 95 (28%) males. The majority of the respondents were in the age group of 31-41 years (37.7%), followed by 30 years (35.1%) and 42-52 years (19.1%). Most participants were non-Saudis (68.4%), worked as a nurse (81.9%), and in the inpatient department (68.9%). In addition, 108 (31.8%) participants worked only for less than two years, followed by two to five years (28.3%) and six to ten years (20.3%). The majority of the participants were either single (47.7%) or married (46.3%) (Table 1).

**Table 1:** Demographics of the participants and sample characteristics. n: frequency; %: percentage

Factors	n (%)
Age (in years)	Total = 339
<30	119 (35.10)
31–41	128 (37.76)
42–52	65 (19.17)
53–60	25 (7.37)
>61	2 (0.59)
Sex	Total = 339
Male	95 (28.02)
Female	244 (71.98)
Nationality	Total = 339
Saudi	107 (31.56)
Non-Saudi	232 (68.44)
Duty (occupation)	Total = 338
Fellow	12 (3.55)
Medical resident	49 (14.50)
Nurse	277 (81.95)
Area	Total = 277
Inpatient	191 (68.95)
Outpatient	86 (31.05)
Length of service (in years)	Total = 339
<2	108 (31.86)
2–5	96 (28.32)
6–10	69 (20.35)
>10	66 (19.47)
Marital status	Total = 339
Divorced or widowed	20 (5.90)
Married	157 (46.31)
Single	1628 (47.79)

Workplace bullying characteristics

The overall score of the NAQ-R scale ranges from 22 to 110. The respondents were classified based on the frequency of their exposure to WPB. Participants were divided into three categories: never bullied (below 33), occasionally bullied (between 33 and 45), and severely bullied (above 45). Although almost half of the participants (46.9%) were categorized into the never bullied category, and the rest were victims of WPB, with 31.8% of participants being occasionally bullied and 21.2% being severely bullied. In general, females were exposed to a higher incidence of WPB compared to their counterparts. Overall, 66.6% of females reported being severely bullied, and 68.52% were occasionally bullied (p = 0.178). In general, respondents in the age group of 31-41 years recorded a significantly higher percentage of WPB, with 48.6% severely bullied and 35.2% occasionally bullied compared to the other age groups. Next on the list were participants aged 30 years or younger, with 38.9% severely bullied and 34.3% occasionally bullied (p = 0.034). Single participants were found to have a higher incidence of severe bullying (48.6%) than married individuals (44.4%), although by just a tiny margin. In comparison, married participants recorded a relatively higher occurrence of occasional bullying (46.3%) compared to single participants (45.3%) (p = 0.598). WPB occurred significantly more in non-Saudis, with percentages ranging from 58.3% severely bullied to 66.7% occasionally bullied compared to Saudis (p = 0.051). Severe bullying was more prevalent in participants with less than two years of experience (31.9%). Occasional bullying took precedence in participants with two to five years of experience (30.6%) (p = 0.609). Among the working groups, nurses were exposed to the majority of severe and occasional bullying with 76.4% and 82.4% of prevalence, respectively, followed by medical residents with 20.8% and 14.8% prevalence, respectively. WPB against fellows was significantly lower with equal percentages (2.8%) for occasional and severe bullying (p = 0.417). There was not much difference among inpatient (54.5%) and outpatient (45.4%) providers when severe bullying was reported. However, inpatient healthcare providers (73%) displayed significant predominance when occasional bullying was disclosed (p = 0.041). R3 residents reported a higher incidence of severe bullying (46.7%) than R1, R2, and R4 residents (p = 0.169) (Table 2).

**Table 2:** Classification of participants based on the modified NAQ-R scale. n: frequency; %: percentage; NAQ-R: Negative Acts Questionnaire-Revised

Factor	Level of bullying				P-value
	n (%)				
	Never bullied	Occasionally bullied	Severely bullied	Total	
Sex					0.1785
Female	122 (76.73)	74 (68.52)	48 (66.67)	244 (71.98)	
Male	37 (23.27)	34 (31.48)	24 (33.33)	95 (28.02)	
Age (in years)					0.0342
≤30	54 (33.96)	37 (34.26)	28 (38.89)	119 (35.10)	
31–41	55 (34.59)	38 (35.19)	35 (48.61)	128 (37.76)	
42–52	32 (20.13)	27 (25.00)	6 (8.33)	65 (19.17)	
53–60	16 (10.06)	6 (5.56)	3 (4.17)	25 (7.37)	
>61	2 (1.26)	0 (0.00)	0 (0.00)	2 (0.59)	
Nationality					0.0511
Non-Saudi	118 (74.21)	72 (66.67)	42 (58.33)	232 (68.44)	
Saudi	41 (25.79)	36 (33.33)	30 (41.67)	107 (31.56)	
Marital status					0.5984
Divorced or Widowed	6 (3.77)	9 (8.33)	5 (6.94)	20 (5.90)	
Married	75 (47.17)	50 (46.30)	32 (44.44)	157 (46.31)	
Single	78 (49.06)	49 (45.37)	35 (48.61)	162 (47.79)	
Length of service (in years)					0.6094
<2	54 (33.96)	31 (28.70)	23 (31.94)	108 (31.86)	
2–5	43 (27.04)	33 (30.56)	20 (27.78)	96 (28.32)	
6–10	31 (19.50)	19 (17.59)	19 (26.39)	69 (20.35)	
>10	31(19.50)	25 (23.15)	10 (13.89)	66 (19.47)	
Duty					0.4172
Fellow	7 (4.43)	3 (2.78)	2 (2.78)	12 (3.55)	
Medical resident	18 (11.39)	16 (14.81)	15 (20.83)	49 (14.50)	
Nurse	133 (84.18)	89 (82.41)	55 (76.39)	277 (81.95)	
Residency year					0.1693
R1	5 (27.78)	1 (6.25)	1 (6.67)	7 (14.29)	
R2	3 (16.67)	6 (37.50)	4 (26.67)	13 (26.53)	
R3	3 (16.67)	6 (37.50)	7 (46.67)	16 (32.65)	
R4	7 (38.89)	3 (18.75)	3 (20.00)	13 (26.53)	
Area					0.0414
Inpatient	96 (72.18)	65 (73.03)	30 (54.55)	191 (68.95)	
Outpatient	37 (27.82)	24 (26.97)	25 (45.45)	86 (31.05)	

Job satisfaction

**Figure 1:**
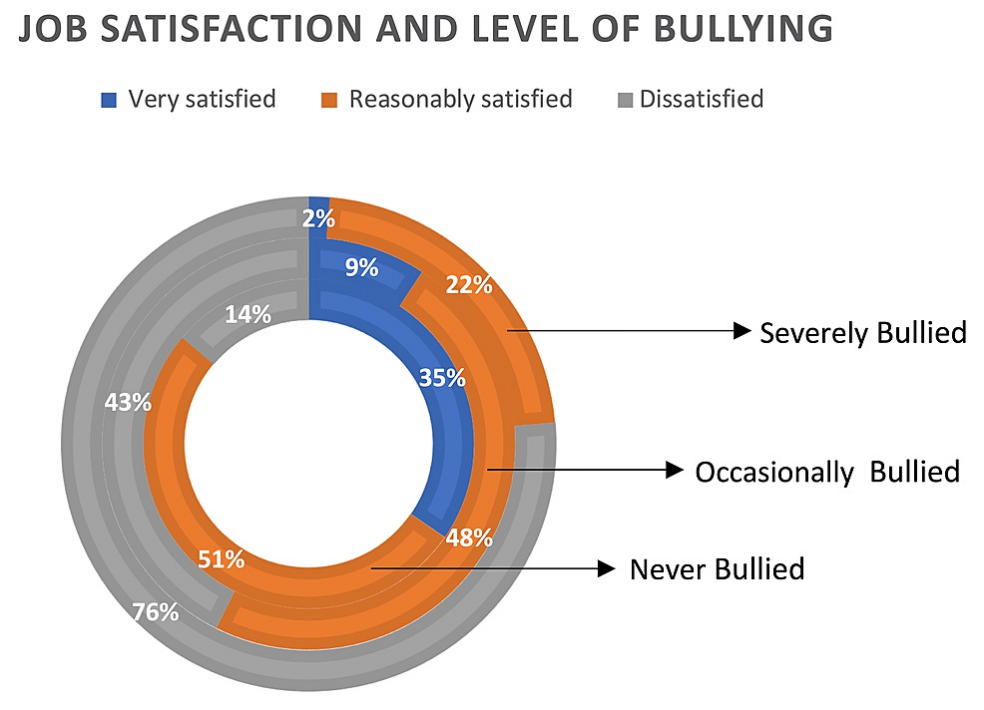
Job satisfaction and the level of bullying.

Most respondents who were never bullied were either very or reasonably satisfied (34.6% and 51.6%, respectively). At the same time, around 42.5% of the occasionally bullied and 76.2% of the severely bullied respondents were little satisfied or dissatisfied. The association of a lower level of satisfaction with a higher level of bullying was statistically significant (p ≤ 0.0001). Overall, 53% reported excellent and 42.1% reported good conditions when asked about their health among the never bullied. Occasionally bullied participants responded with 36.1% excellent and 50.1% good health conditions. Among the severely bullied participants, a low proportion of respondents (33.3%) reported good health conditions (p ≤ 0.0001). When responding to the mental health assessment question, most of the never bullied participants reported it as excellent (48.4%) and good (41.5%). There was a significant association between a decrease in mental health conditions and an increase in bullying levels. Among the occasionally bullied participants, 30.6% reported excellent and 38.9% reported good mental health. The severely bullied participants responded diffusely on a broader spectrum, with 18% excellent, 22.2% good, 19.4% poor, and 13.9% very poor mental health conditions (p ≤ 0.0001) (Table 3, Figure 1).

**Table 3:** Job satisfaction, health, and mental health of the participants correlated with the NAQ-R classification. n: frequency; %: percentage; NAQ-R: Negative Acts Questionnaire-Revised

Factor	Level of bullying				P-value
	n (%)				
	Never bullied	Occasionally bullied	Severely bullied	Total	
How do you feel regarding your work?					<0.0001
Very satisfied	55 (34.59)	10 (9.26)	1 (1.39)	66 (19.47)	
Reasonably satisfied	82 (51.57)	52 (48. 15)	16 (22.22)	150 (44.25)	
A little satisfied	18 (11.32)	37 (34.26)	25 (34.72)	80 (23.60)	
Dissatisfied	3 (1.89)	7 (6.48)	20 (27.78)	30 (8.85)	
Very dissatisfied	1 (0.63)	2 (1.85)	10 (13.89)	13 (3.83)	
How is your health?					<0.0001
Excellent	85 (53.46)	39 (36.11)	24 (33.33)	148 (43.66)	
Good	67 (42.14)	55 (50.93)	24 (33.33)	146 (43. 07)	
Fair	6 (3.77)	11 (10.19)	13 (18.06)	30 (8.85)	
Poor	0 (0.00)	2 (1.85)	7 (9.72)	9 (2.65)	
Very poor	1 (0.63)	1 (0.93)	4 (5.56)	6 (1.77)	
How is your mental health?					<0.0001
Excellent	77 (48.43)	33 (30.56)	13 (18.06)	123 (36.28)	
Good	66 (41.51)	42 (38.89)	16 (22.22)	124 (36.58)	
Fair	14 (8.81)	22 (20.37)	19 (26.39)	55 (16.22)	
Poor	2 (1.26)	10 (9.26)	14 (19.44)	26 (7.67)	
Very poor	0 (0.00)	1 (0.93)	10 (13.89)	11 (3.24)	

## Discussion

This study aimed to investigate bullying behavior against healthcare workers and its effect on job satisfaction, general health, and mental health in a tertiary care hospital. We found that younger females, nurses, non-Saudis, and those working in inpatient settings were more likely to be bullied at work. Furthermore, severely bullied participants were likely to have lower job satisfaction and mental health.

Among the three working classes of healthcare practitioners assessed in this study, the prevalence of WPB was the highest among nurses, with 76.4% reporting severe bullying and 82.4% reporting occasional bullying compared to their counterparts. This might be attributed to their involvement in patient care more often than residents or fellows [6]. The language barrier is an essential factor that exacerbates WPB in the healthcare setting as 82% of the nurses in the study were non-Saudis. Moreover, the limiting of nurses to medical procedures concerning patient interaction further decreases the need for proper communication. Bullying from patients and supervisors was reported to have a higher impact on nurses’ productivity than bullying from physicians and coworkers [11].

On the other hand, physicians interact and communicate more with patients/families regarding their diagnosis, investigations, treatment, and prognosis. Among fellows and residents, bullying from patients or their families is often blamed to be because of an error in this chain of communication with regard to the patients’ health [2]. Another crucial factor for the high prevalence of reported bullying among nurses is gender inequality. Females dominated the nursing sector in the hospital with an 83% prevalence rate compared to their counterparts interspersed in different clinics and departments. Consequently, the rate of severe bullying and occasional bullying reported by females was much higher than males. This finding is consistent with a similar study from the United States where females reported ignoring their opinions, deficient recognition of the efforts and positive results, deprivation of their rights, withholding information, restricted learning opportunities, and overwhelming workload. Moreover, women were more likely to be exposed to repeated incidents of WPB than men [8,22].

Although relatively inexperienced medical personnel have been implicated to be affected by WPB more than the experienced personnel due to their lack of communication skills, inability to form valuable relationships, ineffective coping mechanisms, or working beyond their capabilities, our study did not find a significant difference among the different experience groups [2,9]. The prevalence rate was distributed identically, with a slight predominance in those with less than five years of experience. The rate of WPB was noted to be higher in younger and middle-aged participants till 41 years of age. This high prevalence in the younger age group could be due to being new to the working life and relative inexperience in resisting harmful incidents, as reported by Duru et al. [23].

The incidence of occasional (66.7%) and severe bullying (58.3%) was more frequent among non-Saudis than Saudi healthcare workers. The most dominant factor for this discrepancy was the language barrier and variation in culture and belief [19]. The main language used by medical professionals in the healthcare setting is English, but most patients admitted to our hospital converse in Arabic as their preferred language of communication. Often healthcare workers communicate with their patients in a simple and informal dialect, with a lack of correct reception. This disparity raises the potential for a possible communication error, magnifying a potential WPB incidence. There is a deficiency in the literature correlating WPB incidence to cultural and language differences in a healthcare setting involving patients/their families and healthcare providers [2].

WPB often has consequences that are reflected upon victims. WPB victims often display various symptoms that are often subtle and not recognizable. These effects eventually can jeopardize patient care, have detrimental effects on work performance, decrease the self-esteem and confidence to interact with other healthcare providers, and ultimately put the patients’ safety and other colleagues at stake, predisposing them to medical errors [1,24,25]. WPB is also associated with increased susceptibility to performance decline, vulnerable job positions, and increased professional staff turnover [26]. To assess the aforementioned, participants in our study were asked about their satisfaction with their work in the institute. The majority of the respondents in the severely bullied group were reasonably satisfied to dissatisfied. At the same time, participants in the occasionally bullied group were reasonably satisfied and a little satisfied.

Further questions were asked to assess the physical and mental health of the participants. According to the literature, WPB is associated with somatic complaints such as decreased self-control, burnout, musculoskeletal ailments, higher cardiovascular disease risk, recreational drug abuse, and suicidal attempts [10]. When asked about their health condition, many participants categorized as severely bullied reported either good or excellent. Almost half of the occasionally bullied participants reported their health to be good. The participants’ mental health status question was also asked to evaluate the presence of anxiety, depression, and low self-esteem as an aftermath of WPB [10]. Responses were diverse ranging from excellent to very poor among the severely bullied individuals. Whereas, as expected, a majority in the never bullied group reported their health and mental health conditions to be excellent.

## Conclusions

In this study, we recorded the prevalence, identified the most commonly targeted demographics, and assessed the impact of WPB on healthcare providers at a tertiary care institute located in the capital city of Saudi Arabia. According to the survey results, 53% of healthcare practitioners in different services have experienced some form of WPB. Furthermore, female gender, young age, nurses, and non-Saudi practitioners were the most commonly affected individuals in the medical facility. WPB in any facility is an unfortunate event, mainly in a healthcare setting. WPB affects practitioners physically and mentally, decreasing their maximum potential output and ultimately threatening the quality of care being provided to the patients.

Hence, it is crucial that any incidence of WPB is reported to higher authorities, the root cause of the problem be investigated, and robust actions be taken. In addition, the hospital administration should address the issue of WPB in a more holistic manner. Educating the staff, training the vulnerable groups, monitoring the workplace, and developing anti-WPB policies are some of the strategies that should be established in a healthcare facility to curb the occurrence of WPB.

## Appendices

**Table 4:** The revised NAQ-R scale. NAQ-R: Negative Acts Questionnaire-Revised

	Never	Occasionally	Monthly	Weekly	Daily	
1. Have you been exposed to someone withholding information that affects your performance?						
						2. Have you been humiliated (embarrassed) or ridiculed (make fun of) in connection with your work?						
3. Have you been asked to do work that does not fit your level of competence?						
4. Have you been exposed to having your key areas of responsibility removed or replaced with more trivial or unpleasant tasks?						
5. Have you been exposed to other staff spreading gossip and rumors about you?						
6. Have you been ignored or excluded?						
7. Have you been exposed to insulting or offensive remarks made about your person, your attitudes, or your private life?						
8. Have you been exposed to being shouted at or being the target of spontaneous anger?						
9. Have you been exposed to Intimidating behaviors such as finger-pointing, invasion of personal space, shoving (pushing), blocking your way?						
10. Have you received hints or signals from others that you should quit your job?						
11. Have you been exposed to repeated reminders of your errors or mistakes?						
						12. Have you been exposed to being ignored or facing a hostile reaction when you approach?						
13. Have you been exposed to persistent criticism of your errors or mistakes?						
14. Have you been exposed to having your opinions ignored?						
15. Have you been exposed to practical jokes carried out by people you don’t get along with?						
16. Have you been given tasks which are inappropriate?						
17. Have you been exposed to having allegations made against you?						
18. Were you exposed to excessive monitoring of your work?						
19. Have you had pressure not to claim something to which by right you are entitled?						
20. Have you been exposed to being the subject of excessive teasing and sarcasm?						
21. Have you been exposed to an unmanageable workload?						
22. Have you been exposed to threats of violence?						
23. Have you been exposed to physical abuse or actual abuse?						
